# Supplementary material for: Inhibition of Multifunctional Protein p32/C1QBP Promotes Cytostatic Effects in Colon Cancer Cells by Altering Mitogenic Signaling Pathways and Promoting Mitochondrial Damage
Source: Int J Mol Sci. 2024 Feb 27;25(5):2712. doi: 10.3390/ijms25052712 (PMC10931692; doi:10.3390/ijms25052712)
Supplement: Supplementary file 1 [file ijms-25-02712-s001.zip › Supplementary Figure S1.pdf]

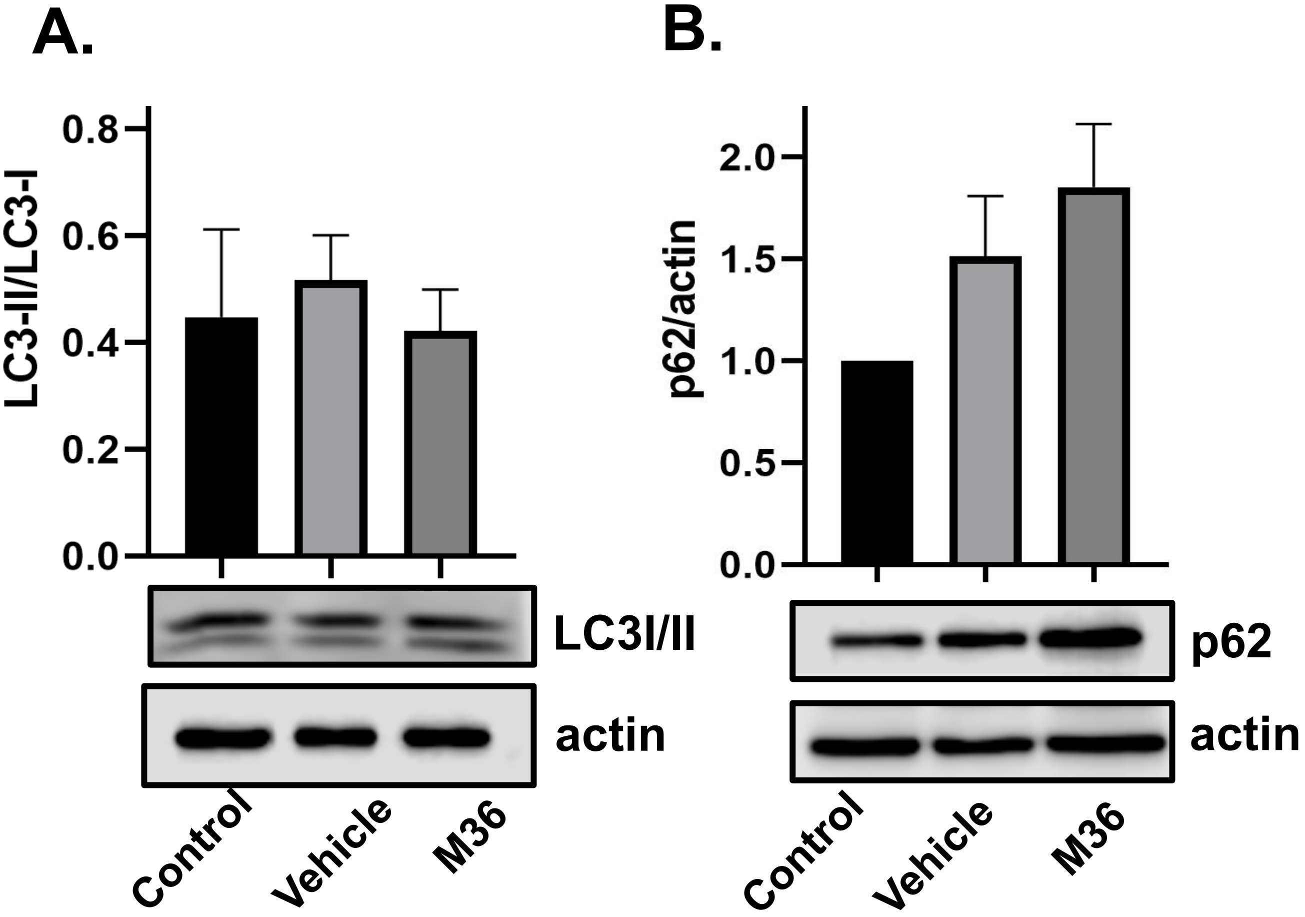

Supplementary Figure S1. Pharmacological inhibition of p32 does not induce macroautophagy in colon cancer cells. RKO cells were treated for 72 h in the absence (control media or DMSO vehicle) or in the presence of the specific p32 inhibitor M36 at the IC50 = 56 μM. The expression of the autophagy markers LC3II/LC3I ratio **(A)** and p62 protein levels **(B)** were then examined in cells byWestern blotting. Actin antibody was used to control for equal loading. Densitometric analysis was performed to estimate the changes in LC3II/LC3I ratio levels and in p62 levels in control or treated RKO cells. Graphs represent the mean values ± SEM from 3 independent experiments.
