# Supplementary material for: Inhibition of Multifunctional Protein p32/C1QBP Promotes Cytostatic Effects in Colon Cancer Cells by Altering Mitogenic Signaling Pathways and Promoting Mitochondrial Damage
Source: Int J Mol Sci. 2024 Feb 27;25(5):2712. doi: 10.3390/ijms25052712 (PMC10931692; doi:10.3390/ijms25052712)
Supplement: Supplementary file 1 [file ijms-25-02712-s001.zip › Supplementary Table S1.pdf]

Supplementary Table S1. Antibodies used in this study.

Antibody	Company	No.	Dilution
Rabbit anti-Akt	Santa Cruz Biotechnology	sc 8312	3000
Mouse anti-Phospho-Akt	Santa Cruz Biotechnology	sc 293125	3000
Mouse anti-DRP1	Santa Cruz Biotechnology	sc-271583	1000
Mouse anti-OPA1	Santa Cruz Biotechnology	sc-393296	1000
Mouse anti-Parkin (PRK8)	Santa Cruz Biotechnology	sc-32282	500
Rabbit anti-cleaved PARP	Cell Signaling Technology	#5625	500
Rabbit anti-cleaved Caspase-3	Cell Signaling Technology	#9664	2000
Rabbit anti-p21	Cell Signaling Technology	#2947	1000
Rabbit anti-phospho-mTOR	Cell Signaling Technology	#2971	3000
Rabbit anti-mTOR	Cell Signaling Technology	#2983	3000
Mouse anti-Phospho-p70 S6 Kinase	Cell Signaling Technology	#9206	3000
Rabbit anti-p70 S6 kinase	Cell Signaling Technology	#9202	3000
Rabbit anti-Phospho ERK1/2	Cell Signaling Technology	#9101	3000
Rabbit ERK 1/2	Cell Signaling Technology	#4695	3000
Rabbit anti-4E-BP1	Cell Signaling Technology	#9644	2000
Rabbit anti-Phospho-4E-BP1	Cell Signaling Technology	#2855	2000
Rabbit anti-Phospho-ULK1 (Ser757)	Cell Signaling Technology	#68885	2000
Rabbit anti-Pink1 (D3G3)	Cell Signaling Technology	#6946	1000
Rabbit anti-SQSTM1/p62	Cell Signaling Technology	#8025	1000
LC3B/MAP1LC3B	Novus	NB100-220	5000
FITC-conjugated goat anti-mouse	Jackson ImmunoResearch	#115-095-003	200
Goat anti-mouse IgG-horseradish peroxidase-conjugate	R & D	#HAF 007	4000
Goat anti-rabbit IgG-horseradish peroxidase-conjugate	R & D	#HAF 008	4000
Mouse anti-p32 (gC1qR)	Abcam	#24733	3000
Mouse anti-Mitofusin-2	Abcam	#56889	1000
Mouse anti-actin	Donation from Dr. José Manuel Hernández		4000
