# Supplementary figures and images for: Inhibition of Multifunctional Protein p32/C1QBP Promotes Cytostatic Effects in Colon Cancer Cells by Altering Mitogenic Signaling Pathways and Promoting Mitochondrial Damage
Source: Int J Mol Sci. 2024 Feb 27;25(5):2712. doi: 10.3390/ijms25052712 (PMC10931692; doi:10.3390/ijms25052712)

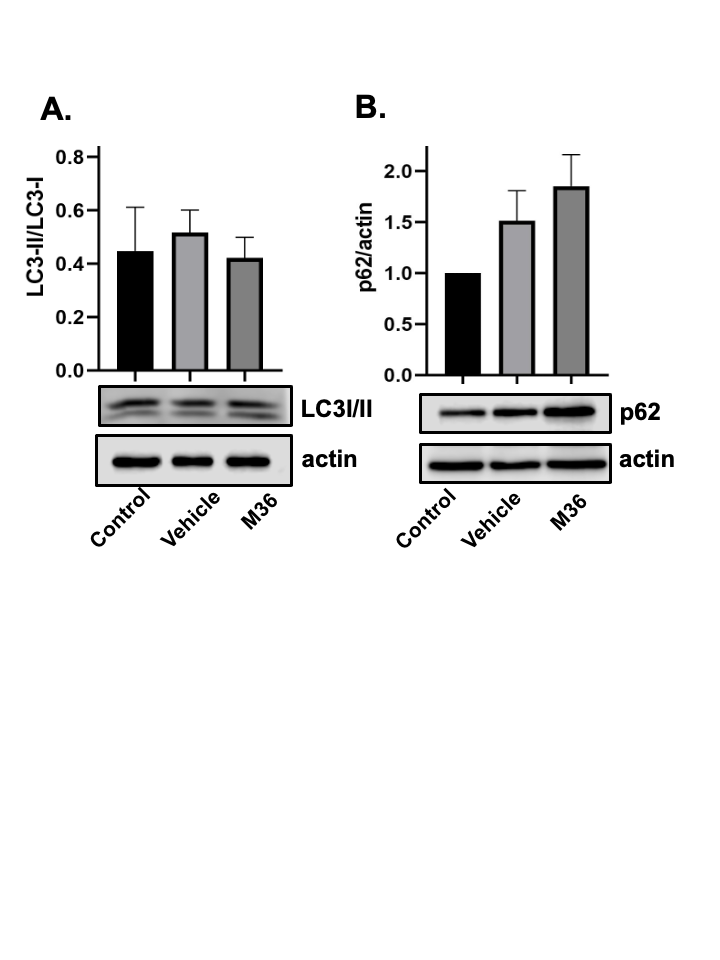

Supplement: Supplementary file 1 [file ijms-25-02712-s001.zip › Supplementary FIgure S1 HQ.tif]
